# Protocol for stromal vascular fraction isolation from inguinal subcutaneous white adipose tissue and beige adipocyte differentiation

**DOI:** 10.1016/j.xpro.2025.104115

**Published:** 2025-09-26

**Authors:** Qiqi Tang, Juan Xu, Xinyu Yang, Hanni Li, Lingdi Wang, Lu Zhu

**Affiliations:** 1 Department of Pharmacology, State Key Laboratory of Experimental Hematology, Tianjin Key Laboratory of Inflammatory Biology, The Province and Ministry Co-sponsored Collaborative Innovation Center for Medical Epigenetics, NHC Key Laboratory of Hormones and Development, Chu Hsien-I Memorial Hospital and Tianjin Institute of Endocrinology, School of Basic Medical Sciences, Tianjin Medical University, Tianjin, China; 2 Department of Physiology and Pathophysiology, Tianjin Key Laboratory of Cell Homeostasis and Major Diseases, School of Basic Medical Sciences, Tianjin Medical University, Tianjin, China

**Keywords:** Cell culture, Cell isolation, Cell Differentiation

## Abstract

Research on inducing beige adipocytes to combat obesity and its comorbidities is growing, with stromal vascular fraction (SVF) from white adipose tissue showing potential to differentiate into both white and beige adipocytes. Here, we present a protocol for isolating the SVF from the iWAT (inguinal white adipose tissue) of mice and differentiating them into mature beige adipocytes. This protocol can be used to analyze the translational regulation and metabolic processes of beige adipocytes *in vitro*.

For complete details on the use and execution of this protocol, please refer to Xu et al.^1^

## Highlights

• Steps for dissection of inguinal white adipose tissue from mice
• Procedures for isolation of stromal vascular fraction (SVF) from white adipose tissue
• Instructions for differentiating SVF into mature beige adipocytes

## Abstract (editor highlights)

Publisher’s note: Undertaking any experimental protocol requires adherence to local institutional guidelines for laboratory safety and ethics.

## Graphical abstract

**Figure:**
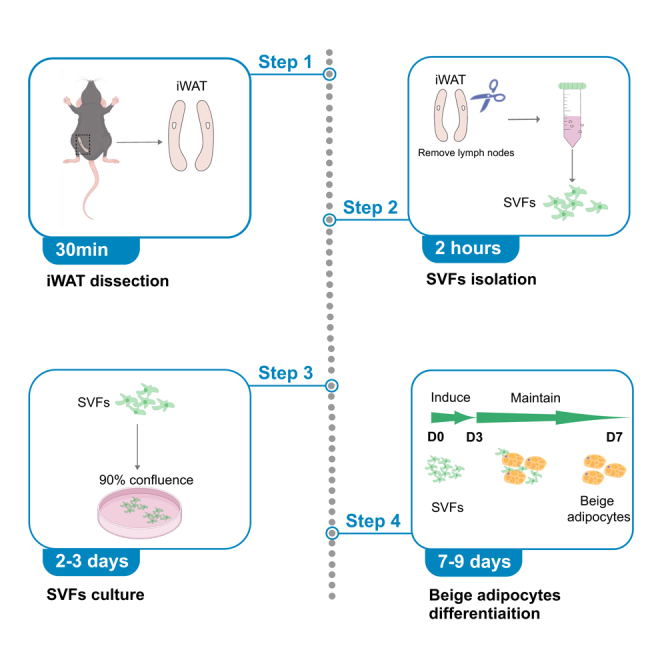

## Before you begin

### Innovation

We specifically focus on the differentiation of SVF derived from iWAT into beige adipocytes. Experiments are designed more precisely according to the characteristics of beige adipocytes, which avoids the interference from brown adipocyte research and improves the specificity of the experiment. The method is simple and convenient for practical operation. Confocal microscopy and Oil Red O staining clearly demonstrate the process of SVF differentiating into beige adipocytes. In addition, the traditional protocol involves a 2-day induction, while we can significantly enhance the differentiation efficiency of beige adipocytes by extending the induction period. In terms of medium maintenance, we adopt half-medium change to reduce the impact of environmental fluctuations on cells and further optimize the differentiation efficiency. Meanwhile, compared with the (250 μm→40 μm) nylon mesh filters used in other literatures, we use 100 μm→70 μm nylon mesh filters, which are more suitable for the cell size of SVF, reducing impurity residues and improving cell purity. Moreover, Red blood cell lysis is set as a mandatory step (4°C for 3–5 min). Through standardized operation, red blood cell contamination is reduced, and the subsequent differentiation efficiency is improved. It is clearly specified that “inoculation in a 6-well plate requires iWAT tissues from 3–4 mice”, which quantifies the demand for starting materials and solves the problem of ambiguous cell seeding density.

The protocol below describes the specific steps for isolating and culturing SVFs from iWAT (inguinal white adipose tissue) as well as differentiating them into mature beige adipocytes expressing uncoupling protein 1 (UCP1). In addition, if mice are used in the experiments, an approved animal use protocol must also be followed.

### Institutional permissions

All animal protocols were in accordance with Institutional Guidelines and approved by the Animal Care and Use Committee of Tianjin Medical University (number: TMUaMEC 2024049). The cell culture study was approved by the Department of Pharmacology, Tianjin Key Laboratory of Inflammatory Biology, School of Basic Medical Sciences, Tianjin Medical University. All mice were on a C57BL/6J background and 4–8 weeks old male or female mice were used for SVFs isolation. The mice were fed with standard normal-chow-diet (NCD) and housed in a specific-pathogen-free facility with a 12-h light-dark cycle and given free access to food and water. The facility temperature and humidity were constantly kept at 22°C and 50%.

### Preparation of consumables and surgery tools

1. For organ dissection.
  a. Prepare scissors and forceps (autoclaved).
  b. Place 75% ethanol for disinfection.
  c. Prepare 50 mL and 15 mL Falcon tubes.
2. For cell isolation and differentiation.
  a. Place the sterilized and pre-cooled PBS as well as the pre-warmed DMEM on the lab bench in advance.
  b. Make a digestion buffer composed of DMEM containing 1 mg/mL collagenase type I (sterilized by 0.22 μm filter).
  c. Prepare 6-well plates are needed for SVF cell seeding and differentiation.
  d. Make a culture medium as DMEM containing 10% fetal bovine serum and 1% penicillin/streptomycin.

***Note:*** Culture medium are stored at 4°C and pre-warmed before usage. PBS (phosphate-buffered saline) should be pre-cooled (4°C). Digestion buffer is prepared freshly prior to usage.

## Key resources table

**Table:** 

REAGENT or RESOURCE	SOURCE	IDENTIFIER
**Antibodies**		

Actin (dilution 1:1,000)	Cell Signaling Technology	Cat# 4967S
UCP1 (dilution 1:1,000)	Abcam	Cat# A10983

**Chemicals, peptides, and recombinant proteins**		

Dexamethasone	Solarbio	Cat# D8040
IBMX	Solarbio	Cat# I8450
Indomethacin	Solarbio	Cat# II0010
T3	Sigma-Aldrich	Cat# T-074
Rosiglitazone	Sigma-Aldrich	Cat# R2408
Insulin	MedChemExpress	Cat# 11061-68-0
Nile Red	Solarbio	Cat# IN0170
DMEM	Meilunbio	Cat# MA0212
FBS (fetal bovine serum)	ExCell	Cat# FSP500
Pen/Strep (penicillin-streptomycin)	MCE	Cat# HY-K1006
75% ethanol	N/A	N/A
Collagenase type 1	Worthington Biochemical	Cat# LS004196
Red blood cell lysis buffer	Solarbio	Cat# R1010
CL-316,243	Sigma-Aldrich	Cat# C5976
Oil red O	Sigma-Aldrich	Cat# O0625

**Experimental models: Organisms/strains**		

Mouse: WT: C57BL/6J (4–8 weeks, male)	Shanghai Laboratory Animal Center (SLAC)	N/A

**Oligonucleotides**		

Ucp1 Fw GAGGTCGTGAAGGTCAGAAT	N/A	N/A
Ucp1 Rv CTGTGGTGGCTATAACTCTGTAA	N/A	N/A
Prdm16 Fw CAGCACGGTGAAGCCATTC	N/A	N/A
Prdm16 Rv GCGTGCATCCGCTTGTG	N/A	N/A
Cidea Fw GGTGGACACAGAGGAGTTCTTTC	N/A	N/A
Cidea Rv CGAAGGTGACTCTGGCTATTCC	N/A	N/A
Dio2 Fw CAGTGTGGTGCACGTCTCCAATC	N/A	N/A
Dio2 Rv TGAACCAAAGTTGACCACCAG	N/A	N/A
Ppargc1α Fw AGCCGTGACCACTGACAACGA	N/A	N/A
Ppargc1α Rv GCTGCATGGTTCTGAGTGCT	N/A	N/A
Fabp4 Fw GCAGAGCCAGGAGAACTTTG	N/A	N/A
Fabp4 Rv GGGTCCATAGGTGATGGTGAG	N/A	N/A
Adipoq Fw AGATGGCACTCCTGGAGAGAAG	N/A	N/A
Adipoq Rv ACATAAGCGGCTTCTCCAGGCT	N/A	N/A
36B4 Fw AGATTCGGGATATGCTGTTGGC	N/A	N/A
36B4 Rv TCGGGTCCTAGACCAGTGTTC	N/A	N/A

**Software and algorithms**		

Adobe Illustrator 27.5	Adobe	https://www.adobe.com/cn/products/illustrator.html
Figdraw	Figdraw	https://www.figdraw.com/static/index.html#/

**Other**		

0.22 μm filter	Guangzhou Jet Bio-Filtration Co., Ltd.	Cat# FPE204030
70 μm nylon mesh	Guangzhou Jet Bio-Filtration Co., Ltd.	Cat# CSS013070
100 μm nylon mesh	Guangzhou Jet Bio-Filtration Co., Ltd.	Cat# CSS013100
6-well cell culture plate (optional)	Guangzhou Jet Bio-Filtration Co., Ltd.	Cat# TCP011006
15 mL tube	Guangzhou Jet Bio-Filtration Co., Ltd.	Cat# CFT011150
50 mL tube	Guangzhou Jet Bio-Filtration Co., Ltd.	Cat# CFT011500
RNeasy mini kit	QIAGEN	Cat# 74104
Confocal laser scanning microscope	Nikon	A1R
CFX Connect real-time PCR detection system	Bio-Rad	CFX96
Surgical scissors	SHINVA	ZC341R/RN
Water bath	Suzhou Jimei Electronic Co., Ltd.	DK-8D

## Materials and equipment

Preparation of buffers and solutions for isolating SVF from mouse iWAT and inducing its differentiation into mature beige adipocytes.

**Table:** PBS

Reagent	Final concentration	Volume/Weight
NaCl	137 mM	8 g
KCl	2.7 mM	0.2 g
Na_2_HPO_4_	10 mM	1.44 g
KH_2_PO_4_	1.8 mM	0.24 g
Total	N/A	1 L

***Note:*** Adjust pH to 7.3, it can be stored at 20°C-25°C. For long-term use (more than 1 month), storage at 4°C is recommended. This solution can be stored at 4°C for a maximum of 6 months.

**Table:** Digestion buffer

Reagent	Final concentration	Volume/Weight
DMEM ( or use Krebs buffer )	N/A	50 mL
Collagenase Type 1	1 mg/mL	50 mg
Total	N/A	50 mL

***Note:*** The digestion buffer should be freshly prepared before use and should not be stored.

**Table:** Induction medium

Reagent	Final concentration	Stock concentration
Insulin^2^	10 μg/mL	10 mg/mL
Rosiglitazone^3^	1 μM	1 mM
3-isobutyl-1-methylxanthine (IBMX)	0.5 mM	0.5 M
Dexamethasone	1 μM	1 mM
Triiodothyronine (T3)	1 nM	1 μM
Indomethacin	1 mM	1 M

***Note:*** Always prepare freshly, do not store.

**Table:** Maintenance medium

Reagent	Final concentration	Stock concentration
Insulin	10 μg/mL	10 mg/mL
Rosiglitazone	1 μM	1 mM
Triiodothyronine (T3)	1 nM	1 μM
Indomethacin	1 mM	1 M

***Note:*** Always prepare freshly, do not store.

## Step-by-step method details

### Dissection of iWAT from mice

**Timing: 30 min**

**CRITICAL:** The initial number of fat depots is important, as it will dictate the seeding density of cell culture which will affect differentiation efficiency. For a 6-well plate you require either at least iWAT depots from 3 to 4 mice, the number of depots can be scaled accordingly.

**Figure 1:**
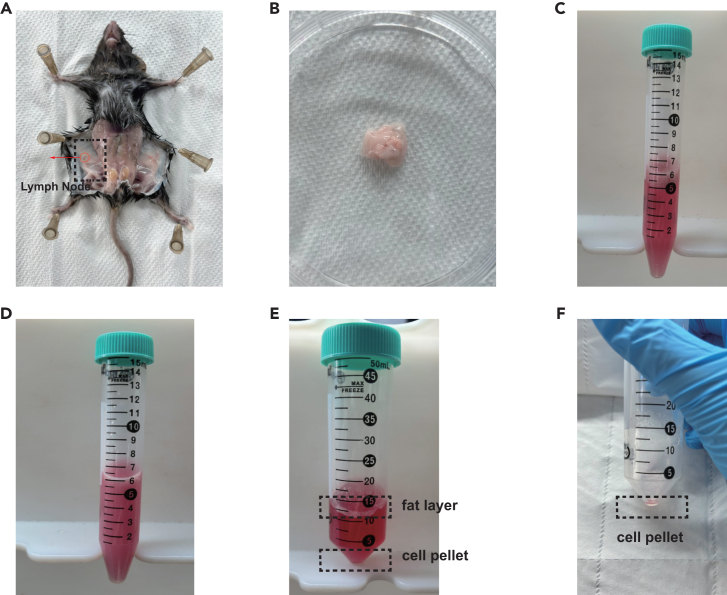
Isolation of SVFs from iWAT (A) Mice anatomical location of inguinal adipose tissue (iWAT). (B) Isolation of iWAT from mice. (C) Transfer tissue pieces into digestion buffer. (D) Digestion buffer with complete digested tissue. (E) Digestion buffer after centrifugation. (F) SVFs pellet.

1. Prepare warm digestion buffer.
2. Euthanize mice via CO_2_ asphyxiation or other institutionally approved methods.
3. Sterilize the abdominal region with 75% ethanol.
4. Dissect inguinal white adipose tissue and rinse each collected tissue with cold PBS.
  a. Remove the remaining connective tissue and inguinal lymph node of iWAT.
  b. Cover with PBS to prevent the tissue from drying out.
  c. process as quickly as possible in a sterile manner (Figures 1A and 1B).

### Isolation of stromal vascular fraction from murine adipose tissue

**Timing: 2 h**

5. Collect enough fat pads into a 2 ml EP tube, add an appropriate amount of cold PBS, and mince adipose tissue into small pieces (<1–2 mm^3^) using scissors. Large pieces will negatively affect the digestion process and result in a low cell yield.
6. Transfer tissue into 15 mL tube and add 6–8 mL of warm digestion buffer to start the digestion reaction (Figure 1C).
7. Place the tube into 37°C water bath pot. Digest adipose tissue pieces by incubating at 37°C for 15 min to 30 min.
  a. Vigorous shaking by hand for 10 s every 5 min facilitates digestion.
  b. Digestion is completed when almost no visible pieces of adipose tissue are left and the mixture becomes homogenous (Figure 1D).

**CRITICAL:** Stop digestion once large visible tissues disappear and digestion buffer become turbid.

***Note:*** An oscillating water bath can be used as an alternative to manual shaking.

8. Add 2–3 mL of culture medium to stop digestion.
9. Filter through a 100 μm nylon mesh^4^ and capture flow through in a new 50 mL tube.
  a. Rinse the tube, in which the digestion was carried out, with 10 mL of PBS.
  b. Filter through the same nylon mesh.
  c. Combine flow through.
10. Filter through a 70 μm nylon mesh^5^ and capture flow through in a new 50 mL tube.
  a. Rinse the tube, in which the digestion was carried out, with 10 mL of PBS.
  b. Filter through the same nylon mesh.
  c. Combine flow through.
11. Centrifuge at 1000 × *g* for 15 min at 4°C (Figure 1E).
12. Remove the fat layer and supernatant, and lyse the red blood cells.
  a. Completely remove fat layer and supernatant (do not disturb the cell pellet).
  b. Add 3–5 ml of red blood cell lysis buffer to gently resuspend the cells.
  c. Let it stand in a 4°C refrigerator for 3–5 min, then add 10 ml of cell culture medium to terminate the lysis.
13. Centrifuge at 1000 × *g* for 15 min at 4°C and remove supernatant (Figure 1F).

**CRITICAL:** To completely remove the fat layer, it is recommended to carefully discard the obvious fat layer on the top first, and then discard the clear liquid on the top; add fresh DMEM to resuspend the cell pellet and centrifuge again is necessary.

14. Resuspend the SVF cell pellet in 1 mL of culture medium.
15. Seed cells in a 6-well plate with 1 mL of culture medium (the total volume per well is 2 ml), and incubate at 37°C with 5% CO2.

### Culture of stromal vascular fraction from murine adipose tissue

**Timing: 2–3 days**

16. Wash cells twice with warm 1% Pen/Strep PBS to remove cell debris the next day.
17. Add fresh culture medium.
18. Change fresh culture medium every day to ensure that the cells are in perfect condition until the cells reach 90% confluency.

**CRITICAL:** It’s crucial to note that the SVF pellet separated from iWAT contains not only primary preadipocytes but also other essential components, including macrophages, and endothelial cells, among others, so it is necessary to wash cells completely next day.

### Beige adipocyte differentiation

**Timing: 7–9 days**

19. Day 0–3: Change growth medium until the cells reach 90% confluency (termed “day 0”). On day 0 remove growth medium and add 2 mL of induction medium per well of 6-well plate.

**CRITICAL:** Change induction medium on day 2 is necessary for that there is more metabolic waste in the culture medium due to the high cell density and vitality.

***Note:*** All are the final working concentrations for cell treatment: 0.5 mM 3-isobutyl-1- methylxanthine (IBMX), 1 μM dexamethasone, 1 μM rosiglitazone, 10 μg/mL insulin, 1 mM indomethacin and 1 nM triiodothyronine (T3).

20. Day 4–9: Replace maintenance medium every 2 days.

**Figure 2:**
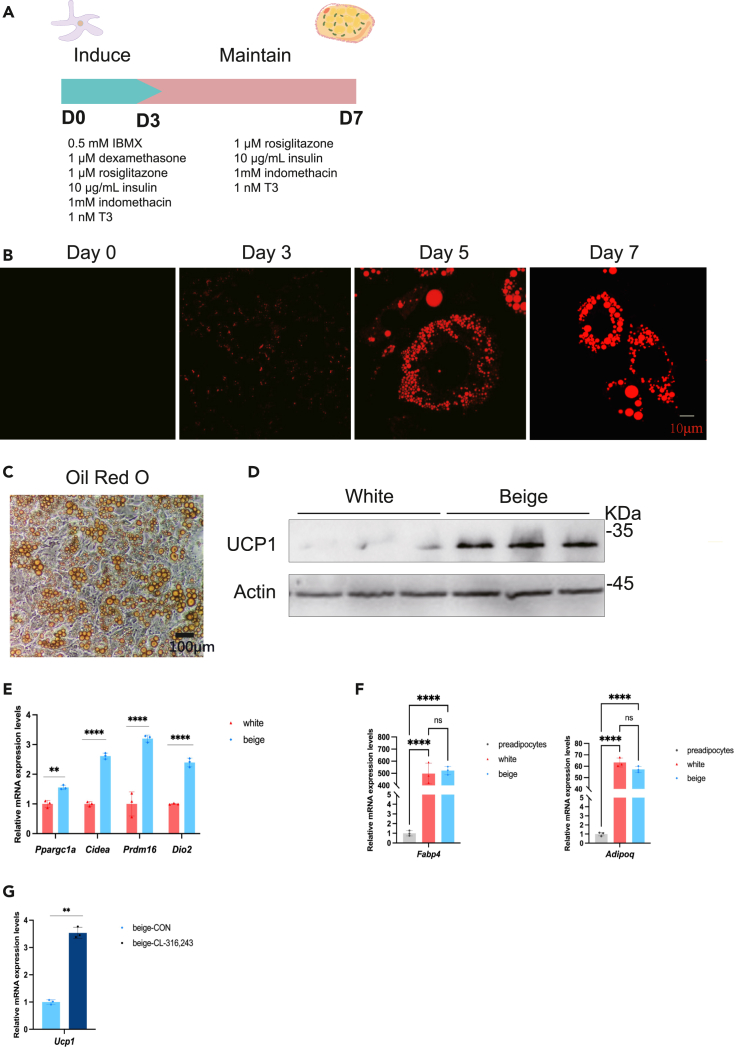
Differentiation of beige adipocytes from SVFs (A) Schematic protocol of beige adipocytes differentiation. (B) Nile Red staining of cells on day 0, 3, 5, 7. Scale bar: 10 μm. (C) Oil Red O staining of SVFs-differentiated mature beige adipocytes. Scale bar: 100 μm. (D) Immunoblotting of UCP1 protein expression in SVFs-differentiated white adipocytes and beige adipocytes. (E) RT-PCR analysis of thermogenic genes in SVFs-differentiated white adipocytes and beige adipocytes. (F) RT-PCR analysis of adipogenesis genes in SVFs-differentiated white adipocytes and beige adipocytes. (G) RT-PCR analysis of *Ucp1* in SVFs-differentiated beige adipocytes with or without 10 μM CL-316,243 stimulation. ∗p < 0.05, ∗∗p < 0.01, ∗∗∗p < 0.001, ∗∗∗∗p < 0.0001, by one-way ANOVA. All data are presented as mean ± SD.

***Note:*** The marker for the differentiation of SVF into mature beige adipocytes is that on days 8–9 of differentiation, the adipocytes are densely filled with lipids, and 70%–90% of the cells contain dense lipid droplets (Figures 2B and 2C).

***Note:*** After beige adipocytes differentiate and mature, adipogenic genes such as Adipoq and Fabp4 can be detected by RT-PCR. A significant fold increase indicates successful differentiation (Figure 2F).

**CRITICAL:** To increase differentiation efficiency, apply method for 3-days induction and 5-days maintenance instead of traditional protocol of 2-days induction is crucial (Figure 2A). For our experiments, we differentiated cells until day 7 to match the in vivo treatment.

***Note:*** All are the final working concentrations for cell treatment:1 μM rosiglitazone, 10 μg/mL insulin, 1mM indomethacin and 1 nM triiodothyronine (T3).

***Note:*** Cells can be differentiated for longer periods if needed. Also, retaining half of the original maintenance medium and adding half of fresh maintenance medium can improve the differentiation efficiency if necessary.

## Expected outcomes

The protocol for isolating SVF and differentiating it into mature beige adipocytes aims for several key outcomes. Upon adipogenic induction SVFs will drastically alter their morphological appearance. Small lipid droplets will appear on day 3 and the number and size of lipid droplets will further increase during differentiation process until adipocytes are densely packed with lipids (Figure 2B). Also, differentiation efficiency, the percentage of cells containing triglycerides in the form of lipid droplets, ranges between 70%–90% (Figure 2C). These beige adipocytes have high-UCP1 expression and significant upregulation of thermogenic genes, along with a multilocular smaller lipid droplet morphology (Figures 2B–2E). The differentiation degrees of white adipocytes and beige adipocytes are similar (Figure 2F), and the method for inducing white adipocytes can be referred to in this literature.^3^ To further strengthen the beige phenotype of the adipocytes obtained, the response of beige adipocytes to a beta-adrenergic agonist (isoproterenol, CL-316,243) or norepinephrine) in term of *Ucp1* expression for example could be analyzed (Figure 2G). We treated the cells with 10 μM CL-316,243 for 1 h.

## Limitations

Beige fat is a potential therapeutic target for obesity and other metabolic diseases due to its inducible brown fat-like functions and enriched mitochondria.^6^^,^^7^ iWAT can undergo robust brown remodeling with appropriate stimuli and is therefore widely considered as a representative beige fat depot.^5^^,^^8^ This protocol describes methods for isolating SVFs and high-efficiently differentiating beige adipocytes. However, since the SVF contains not only adipose precursor cells but also other components such as macrophages and vascular cells, these factors may interfere with cellular phenotypes, making it impossible to fully replicate the in vitro experimental results.^9^^,^^10^ During SVF isolation, we pooled samples from multiple murine fat depots, but individual variability among mice may affect the consistency of subsequent experiments. Additionally, factors such as mouse age, cell density of plating, and initial cell density at the onset of differentiation can significantly impact differentiation efficiency.^11^^,^^12^

## Troubleshooting

### Problem 1

Insufficient SVFs quantity.

Insufficient adipose tissue collection from limited fat depots, coupled with unexpected attenuation of collagenase enzymatic activity during digestion.

### Potential solution

To obtain sufficient SVF, the following measures can be taken: increase the adipose tissue yield by pooling fat depots from more mice, use freshly prepared collagenase to ensure optimal enzymatic activity, extend the digestion incubation time to achieve complete tissue dissociation.

### Problem 2

Cell contamination.

The instruments were not disinfected thoroughly during the dissection process and contamination occurred during the cell culture process.

### Potential solution

To avoid cell contamination, we should use sterile surgical tools (be careful with the fur when dissecting), add 1% penicillin/streptomycin in digestion buffer and process tissue in biosafety cabinet. If contamination occurs, replace it with fresh medium containing 10 μg/mL ciprofloxacin.

### Problem 3

Low differentiation efficiency.

The SVF are derived from too old mice, resulting in a decrease in the differentiation potential of SVF. The density of cells at the beginning of differentiation is inappropriate, the drug is inactivated, or the drug concentration is insufficient.

### Potential solution

To increase differentiation efficiency, we should use 4–8-weeks-old mice for maximal progenitor cell yield, change new drug or increase concentration of induction medium. The cell confluence is approximately 90% when changing to beige adipocyte induction medium. It’s necessary to change to a fresh medium every 2 days. Meanwhile, retaining half of the original maintenance medium and adding half of fresh maintenance medium is optional.

## Resource availability

### Lead contact

Further information and requests for resources or reagents should be directed to the lead contact, Lu Zhu (zhulu@tmu.edu.cn).

### Technical contact

Technical questions on executing this protocol should be directed to and will be answered by the technical contact, Qiqi Tang (tangqq630@163.com).

### Materials availability

This study did not generate new unique reagents.

### Data and code availability

This study did not generate data or code.

## Acknowledgments

This work was supported by the Science & Technology Development Fund of the Tianjin Education Commission for Higher Education (grant no. 2024ZD038, L.Z.).

## Author contributions

J.X. and Q.T. established the SVF isolation and beige adipocyte differentiation; experiments were performed by Q.T., J.X., X.Y., and H.L. The manuscript was written by Q.T., J.X., L.W., and L.Z. with input from all authors.

## Declaration of interests

The authors declare no competing interests.
